# dbAMEPNI: a database of alanine mutagenic effects for protein–nucleic acid interactions

**DOI:** 10.1093/database/bay034

**Published:** 2018-04-02

**Authors:** Ling Liu, Yi Xiong, Hongyun Gao, Dong-Qing Wei, Julie C Mitchell, Xiaolei Zhu

**Affiliations:** 1 School of Life Sciences, Anhui University, Hefei, Anhui 230601, China; 2 State Key Laboratory of Microbial Metabolism and School of Life Sciences and Biotechnology, Shanghai Jiao Tong University, Shanghai 200240, China; 3 Information and Engineering College, Dalian University, Dalian 116622, Liaoning, China; 4 Department of Biochemistry, University of Wisconsin-Madison, Madison, WI 53706, USA; 5 Department of Mathematics, University of Wisconsin-Madison, Madison, WI 53706, USA; 6 Oak Ridge National Laboratory, Biosciences Division, Oak Ridge, TN 37830, USA

## Abstract

Protein–nucleic acid interactions play essential roles in various biological activities such as gene regulation, transcription, DNA repair and DNA packaging. Understanding the effects of amino acid substitutions on protein–nucleic acid binding affinities can help elucidate the molecular mechanism of protein–nucleic acid recognition. Until now, no comprehensive and updated database of quantitative binding data on alanine mutagenic effects for protein–nucleic acid interactions is publicly accessible. Thus, we developed a new database of Alanine Mutagenic Effects for Protein-Nucleic Acid Interactions (dbAMEPNI). dbAMEPNI is a manually curated, literature-derived database, comprising over 577 alanine mutagenic data with experimentally determined binding affinities for protein–nucleic acid complexes. It contains several important parameters, such as dissociation constant (*K_d_*), Gibbs free energy change (*ΔΔG*), experimental conditions and structural parameters of mutant residues. In addition, the database provides an extended dataset of 282 single alanine mutations with only qualitative data (or descriptive effects) of thermodynamic information.

**Database URL**: http://zhulab.ahu.edu.cn/dbAMEPNI

## Introduction

Protein–nucleic acid interactions play essential roles in cellular activities, and they dictate the development of complex multicellular organisms. Although the 3D structures of many protein–nucleic acid complexes have been solved, the general principles governing protein–nucleic acid interactions are not yet fully understood. Alanine scanning mutagenic experiments (1, 2), which measure the effect of alanine substitutions on binding affinity, can be helpful to extrapolate the mechanisms of protein–nucleic acid recognition. Alanine scanning mutagenesis data can provide ‘hotspot’ information on protein–nucleic acids interfaces. Hotspot residues are a small subset of the buried amino acids that contribute the majority of binding affinity when proteins interact with other biomolecules. While hotspots in protein–protein interfaces have been extensively studied (3–5), there is little comprehensive study of hotspots for protein–nucleic acids.

With the increasing availability of protein–nucleic acid complex structures (6–9), it is urgent to construct a centralized repository of alanine scanning data on protein–nucleic acid interfaces. Prabakaran *et al.* (10, 11) built a Thermodynamic Database for Protein–Nucleic Acid Interactions (ProNIT) by collecting thermodynamic data on protein–nucleic acids interactions from published work. However, ProNIT is outdated, as it only contains data published prior to 2012, and ProNIT is not a database specifically for alanine mutation. In this study, we have developed an extensive repository of alanine mutagenic data for protein–nucleic acid interfaces: dbAMEPNI, a database of Alanine Mutagenic Effects for Protein-Nucleic Acids Interaction (http://zhulab.ahu.edu.cn/dbAMEPNI). dbAMEPNI provides alanine mutagenic effects for over 859 mutations in 217 protein–nucleic acid complexes. In addition, dbAMEPNI provides useful structural information such as the solvent accessible surface areas (SASA), secondary structure and hydrogen bonds. We believe that our database will benefit for the study of protein–nucleic acid interactions and provide a useful benchmark dataset for training and testing computational methods to predict hotspots on protein-nucleic acid interfaces.

## Materials and methods

### Data sources

The alanine mutagenic data were collected from two different sources: the first one is the database ProNIT, containing thermodynamics data for protein–nucleic acid interactions which was published before 2012; the second source is literature published between January 2011 and October 2017. All data in our database have corresponding 3D structures of the protein–nucleic acid complexes available in the Protein Data Bank.

### Structural features calculation

We calculated several features of each mutated residue in the database. The SASA of the wild-type residues in both bound and unbound states were calculated by NACCESS (12) (Version 2.1.1 http://www.bioinf.man.ac.uk/naccess/). Both features are based on holo protein–nucleic acids complexes. The secondary structure to each mutation residue was assigned by DSSP (13, 14). Hydrogen bonds formed between the residues and the nucleic acids were determined by WHAT IF (15).

### Website construction

The database was created by the MySQL relational database management system. Its web pages and interfaces were developed using PHP and Javascript. The visualization system of structures of mutation residues on complexes was developed using GLmol, which is a 3D molecular viewer based on WebGL and Javascript.

## Results and discussion

### Data collection

From ProNIT, we considered a total of 345 single mutation residues by alanine scanning taken from the interfaces of protein–nucleic acid complexes with known 3D structures. Among these data, some were duplicated mutants with different *ΔΔG* values. We checked these duplicates and found that they could be attributed to four major factors: (i) The nucleic acids used for measuring the binding affinity were different. For this situation, we selected the value corresponding to the nucleic acid in the 3D complex structure. (ii) The methods for measuring the binding affinity were different. In this case, we considered the data measured by isothermal titration calorimetry method, which is more accurate than other methods. (iii) The temperatures were different. For this situation, we selected the data obtained near 25°C. (iv) The ion concentrations were different. For this situation, we selected the data obtained near 155 mM. For other complicated situations (totally 35 cases), we calculated the average value of the duplicates. In all, we collected 185 unique mutants from ProNIT. The corresponding PDBIDs are listed in Supplementary Table S1.

In addition, we examined 655 articles that reported the 3D complex structures of protein and nucleic acids in PDB database. The search conditions satisfied the following three requirements: the release date was between 2011-01-01 and 2017-10-01, the macromolecule type was chosen as Protein–DNA or Protein–RNA and the X-ray resolution was <2.5 Å. Thus, we obtained a set of PDBIDs, by which we got the corresponding 655 articles. We looked through the articles, by using the following four schemes to identify the alanine mutagenic information: (i) Read the ‘Method’ section of these articles to check if any mutagenic analysis experiments were conducted in the work; (ii) check the tables and figures in both the main text and the supplementary material to look for and obtain the alanine mutagenesis data; (iii) search the words such as ‘mutant’, ‘mutagenesis’, ‘mutated’ and ‘mutation’, and read the corresponding paragraph in case the alanine mutagenesis data are not shown in the tables and figures; (iv) look through the papers to find the text form such as ‘XnumA’, of which X means different residue types in single letter, ‘num’ is the sequence number of the residue in the protein chain and ‘A’ is the single letter of alanine. After locating the position of alanine mutagenic information, we extracted the alanine mutagenic data, including PDBID, residue information, the mutational effects, reference PMID, page number, experimental methods and conditions and so on. We noticed that the thermodynamic effects of some mutants were measured by quantitative values, whereas the others were reported with only qualitative measures to describe their thermodynamic effects. We thus categorized them into two sets, one set with quantitative data about thermodynamic effects of the mutations (Core set), and the other one with only the descriptive effects (Extended set). From these articles, we obtained 392 and 282 alanine mutants with quantitatively thermodynamic effects and descriptive effects, respectively. The corresponding PDBIDs of these data are showed in Supplementary Table S1.

**Figure 1.**
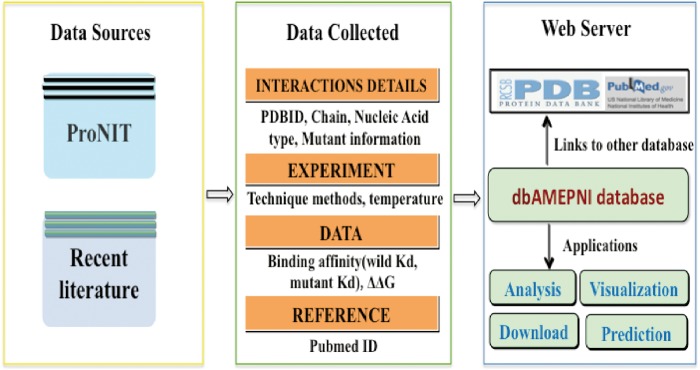
The flowchart for building the dbAMEPNI database.

**Figure 2.**
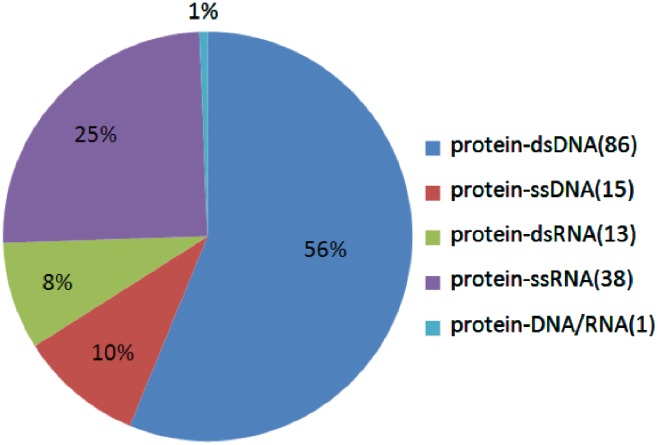
The proportions of the different kinds of protein–NA complexes in the database.

In all, we found 577 alanine mutants with quantitatively characterized thermodynamic effects, along with 282 alanine mutants with qualitatively characterized effects. The flowchart of this process is shown in Figure 1. Furthermore, we analyzed the number of different kinds of interfaces and the number of residues on different kinds of interfaces in the Core Set. As shown in Figure 2, in the Core Set, 101 of the PDB entries are protein–DNA complexes, of which 86 are protein–dsDNA complexes, 15 are protein–ssDNA complexes. 51 of PDB entries are protein–RNA complexes, of which 13 are protein–dsRNA interfaces and 38 are protein–ssRNA complexes. In addition, as shown in Table 1, it was found that 128 residues were from the protein–ssRNA complexes, 65 residues were from the protein–dsRNA complexes, 281 residues were from the protein–dsDNA complexes and 83 residues were from the protein–ssDNA complexes. The remaining complexes and residues are from protein–DNA/RNA. Besides, we also counted number of the hot spot residues in the database, which are 133 in total.

**Table 1.** The numbers of core set residues on different kinds of protein–NA interfaces

Protein–NA interfaces	Number of core set residues
Protein–dsDNA	281
Protein–ssDNA	83
Protein–ssRNA	128
Protein–dsRNA	65
Others	20

### Structural features analysis

**Figure 3.**
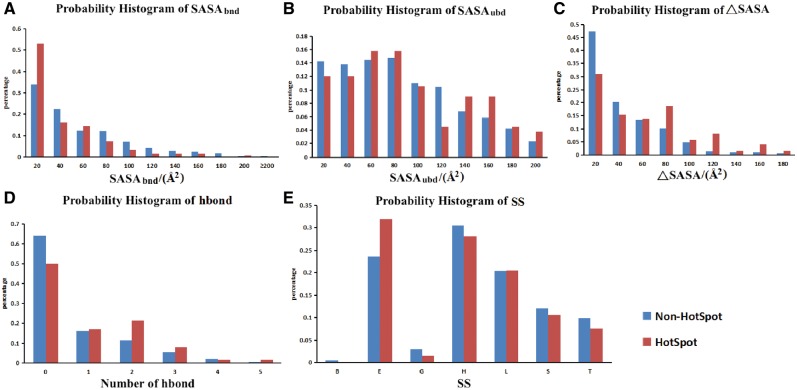
The probability histograms of five features of hotspots and non-hot spots. (**A**) SASA of residues in bound state; (**B**) SASA of residues in unbound state; (**C**) buried SASA (*Δ*ASA); (**D**) hydrogen bond number between proteins and nucleic acids; (**E**) secondary structure (SS). Hot spot was defined with a *ΔΔG* ≥2.0 kcal/mol.

Four structural features were calculated for each mutated residue in the database, including SASAs in both bound and unbound states, secondary structure and hydrogen bond between target residue and nucleic acids. The differences of the four features, along with the buried SASA (ΔSASA), between hotspot residues and non-hot spot residues were analyzed. The buried SASA is the difference between SASAs in unbound and bound states, which was calculated as the following equation:

(1) $$\Delta \text{SASA}={\text{SASA}}_{\text{ubd}}-{\text{SASA}}_{\text{bnd}},$$

where ${\text{SASA}}_{\text{ubd}}$ is the residue’s SASA in unbound state, ${\text{SASA}}_{\text{bnd}}$ is the SASA in bound state. The differences of the five features were analyzed by using an independent *t*-test. The probability (not the frequency) histograms of the five features were shown in Figure 3, to make sure that the *Y*-axis scales of both hot and non-hot spot residues are in the same range. In the figure, the *Y*-axis is the probability of residues in certain feature value/value bin, the *X*-axis is the values of the five features mentioned above. The *t*-test results indicate that the distributions of SASA in bound state and the buried SASA were significantly different between hotspots and non-hotspots, with *P*-values of 0.01 and 0.01, respectively. The average values of SASA in bound state for hot spot residues and non-hotspot residues are 30.93 and 46.79 Å^2^, respectively, which indicates that the hotspot residues on protein–NA interfaces are more inclined to have smaller SASA in bound state than non-hotspot residues. The average values of the buried SASA (*Δ*SASA) for hot spot residues and non-hotspot residues are 51.19 and 31.19 Å^2^, respectively, which indicates that the hotspot residues on protein–NA interfaces are more inclined to have larger *Δ*SASA than non-hotspot residues. This observation is in line with our intuition that the more buried the residue is, the larger probability it is a hotspot residue.

### Data access

dbAMEPNI provides a variety of web-based interfaces and graphical visualizations to facilitate the search and analysis of residues in the database. Users can browse, search and download the data. By the ‘Browse’ web page, users can explore all the entries. On the ‘Browse’ interface, a quick search window can be used to retrieve the entries of interest. By the ‘Search’ web page, users can do some advanced search, for example, users can search entries for which the *ΔΔG* are in a specified range. By the ‘Download’ page, users can download all the data freely on our website.

The website also have a ‘Submit’ web page. We encourage users to submit novel data to the database. Users can submit their data in two ways. First, users can submit a file that contains the new entries, or users can submit their data via a web form. The novel data will be forwarded to the developer and be added to our database after a manual check and confirmation.

The ‘Document’ web page of the website provide a simple tutorial to show how to use the website. It explains the abbreviations in the tables of our database. It also provides a statistical analysis of data along with the years.

**Figure 4.**
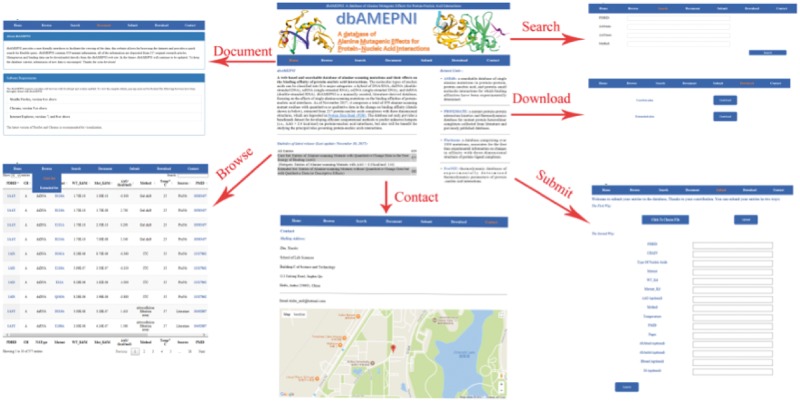
The different interfaces of our website.

**Figure 5.**
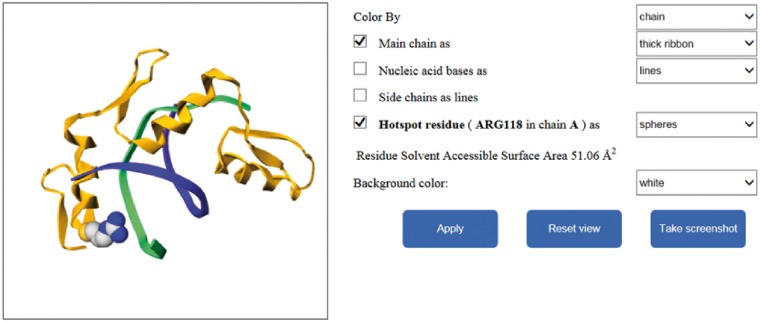
The graphical visualization of the residue in our database.

Different interfaces of the database website are shown in Figure 4. In addition, our website provides additional function to illustrate the mutated residues of protein–nucleic acid complexes by using Gmol. Figure 5 shows an example of the webpage.

### Application of the database

Prior to this alanine mutagenic effect database, there are several studies using the data from ProNIT to analyze the interactions between protein and nucleic acids. Pires *et al.* (16, 17) used the concept of graph-based signatures to predict the effects of the mutations on protein–nucleic acids interfaces. Barik *et al.* (18) analyzed the conservative of the interface residues between protein and RNA, and developed a method to predict the hot spots at protein–RNA recognition sites. Ramos and Moreira (19) developed a computational alanine scanning mutagenesis methodology to predict the hot spots on protein–nucleic acids interfaces. Munteanu *et al.* (20) developed a support vector machine model to predict the hot spots on protein–nucleic acid interfaces based on SASA. The extensive data in our database will help study the hot spots on protein–nucleic acids interfaces and benefit to discover the principals of the interaction between protein and nucleic acids.

### Future perspective

This is the first release of dbAMEPNI database, it contains abundant data of alanine mutagenic effect, which are useful for biochemists and bioinformaticians. In the future, our database will be updated annually based on newly published experimental data. As more data become available, we may divide the database into two parts for protein–DNA and protein–RNA complexes. We will also develop and integrate computational methods for prediction of protein–nucleic acid hotspots into dbAMEPNI.

## Supplementary data

Supplementary data are available at *Database* Online.

## Supplementary Material

Supplementary Table
